# Supplementary figures and images for: PIKI-1, a class II PI 3-kinase, functions in endocytic trafficking
Source: PLoS Genet. 2026 Feb 13;22(2):e1011740. doi: 10.1371/journal.pgen.1011740 (PMC12919929; doi:10.1371/journal.pgen.1011740)

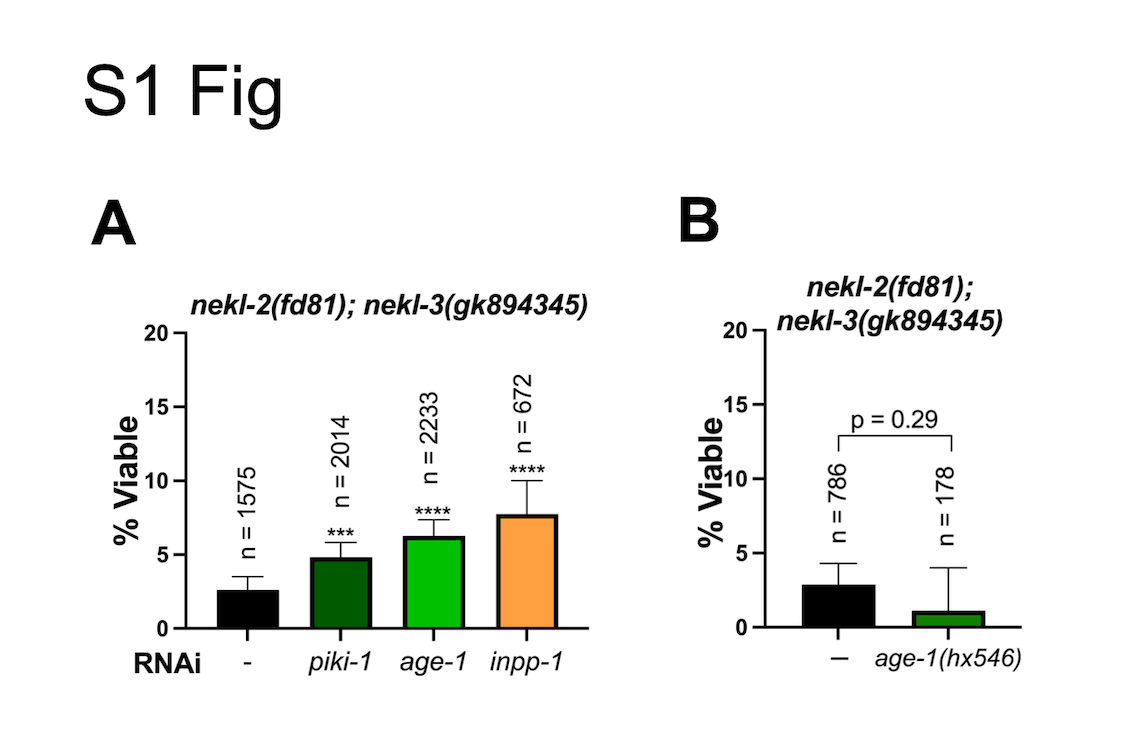

Supplement: S1 Fig — (A) The proportion of viable progeny produced by nekl-2(fd81); nekl-3(gk894345) worms after injection by the indicated dsRNAs. (B) The proportion of viable progeny produced by nekl-2(fd81); nekl-3(gk894345) worms for the indicated genotype. Statistical significance was determined using Fischer’s exact test; ****p ≤ 0.0001, ***p ≤ 0.001. Error bars indicate and 95% CI. Raw data available in this File. (TIFF) [file pgen.1011740.s001.tiff]

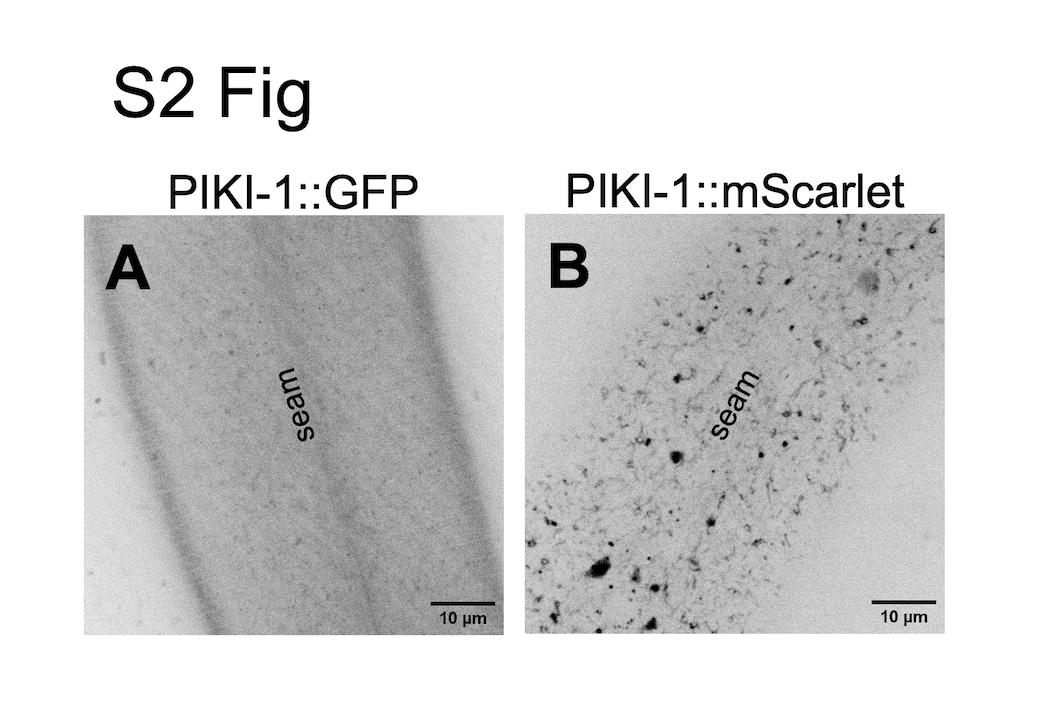

Supplement: S2 Fig — (A, B) Representative confocal images of day-1 adults expressing CRISPR-tagged (A) PIKI-1::GFP and (B) PIKI-1::mScarlet. Note that accumulations present in the PIKI-1::mScarlet strain are likely the result of cleavage of mScarlet and its retention in lysosomes. (TIFF) [file pgen.1011740.s002.tiff]

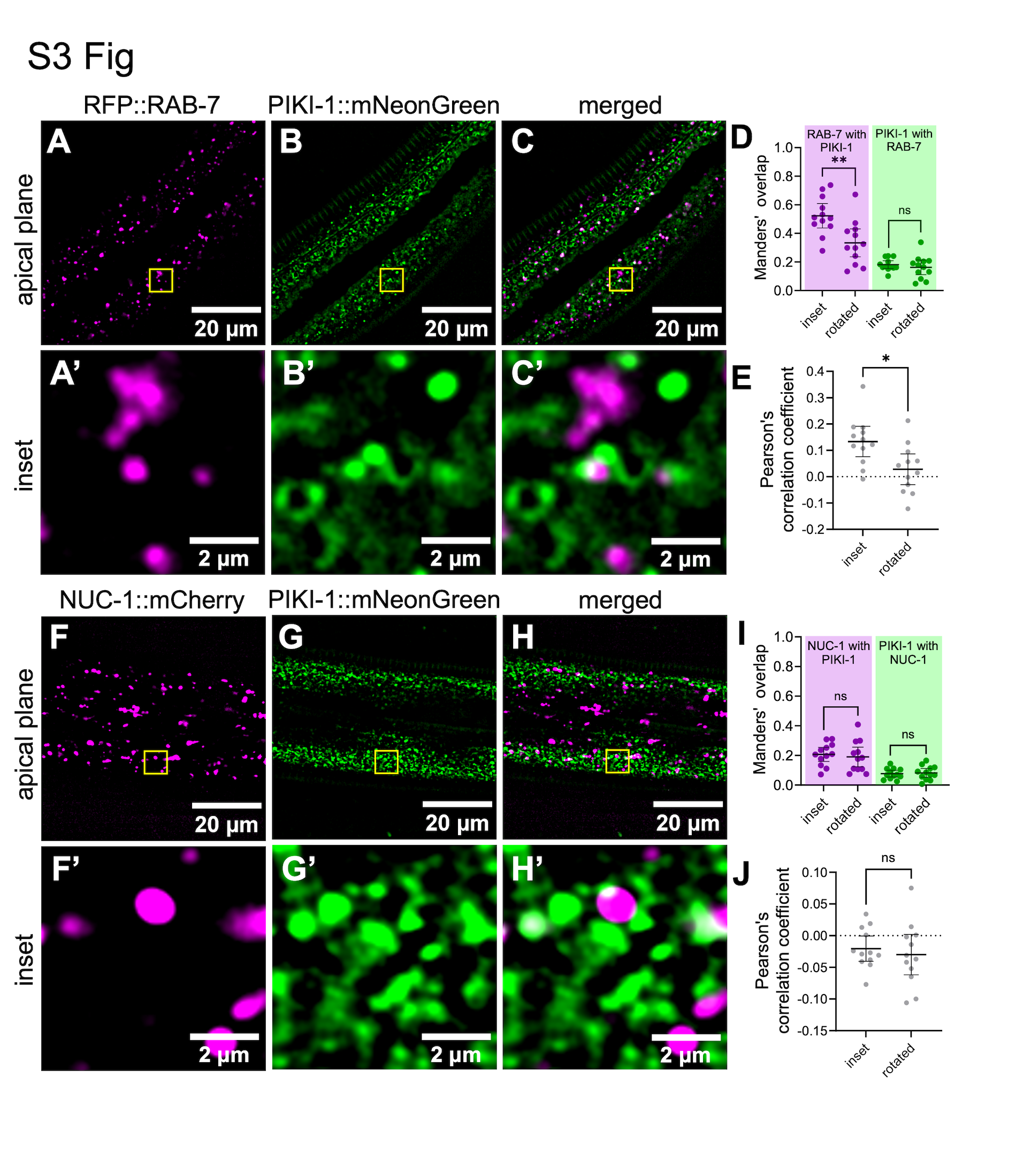

Supplement: S3 Fig — (A–C’ and F–H’) Colocalization of young adult worms expressing Phyp7::PIKI-1::mNeonGreen with (A–C’) GFP::RAB-7 and (F–H’) NUC-1::mCherry. Yellow squares correspond to enlarged insets; yellow arrows indicate examples of colocalization (white). The seam cell is labeled in lower-magnification images. Colocalization was quantified using Mander’s overlap (D, I) and Pearson’s correlation coefficients (E, J). Dot plots show the mean and 95% CI. Statistical significance between rotated and inset values was determined using unpaired t-tests; ns, not significant. Raw data are available in S1 File. (TIFF) [file pgen.1011740.s003.tiff]

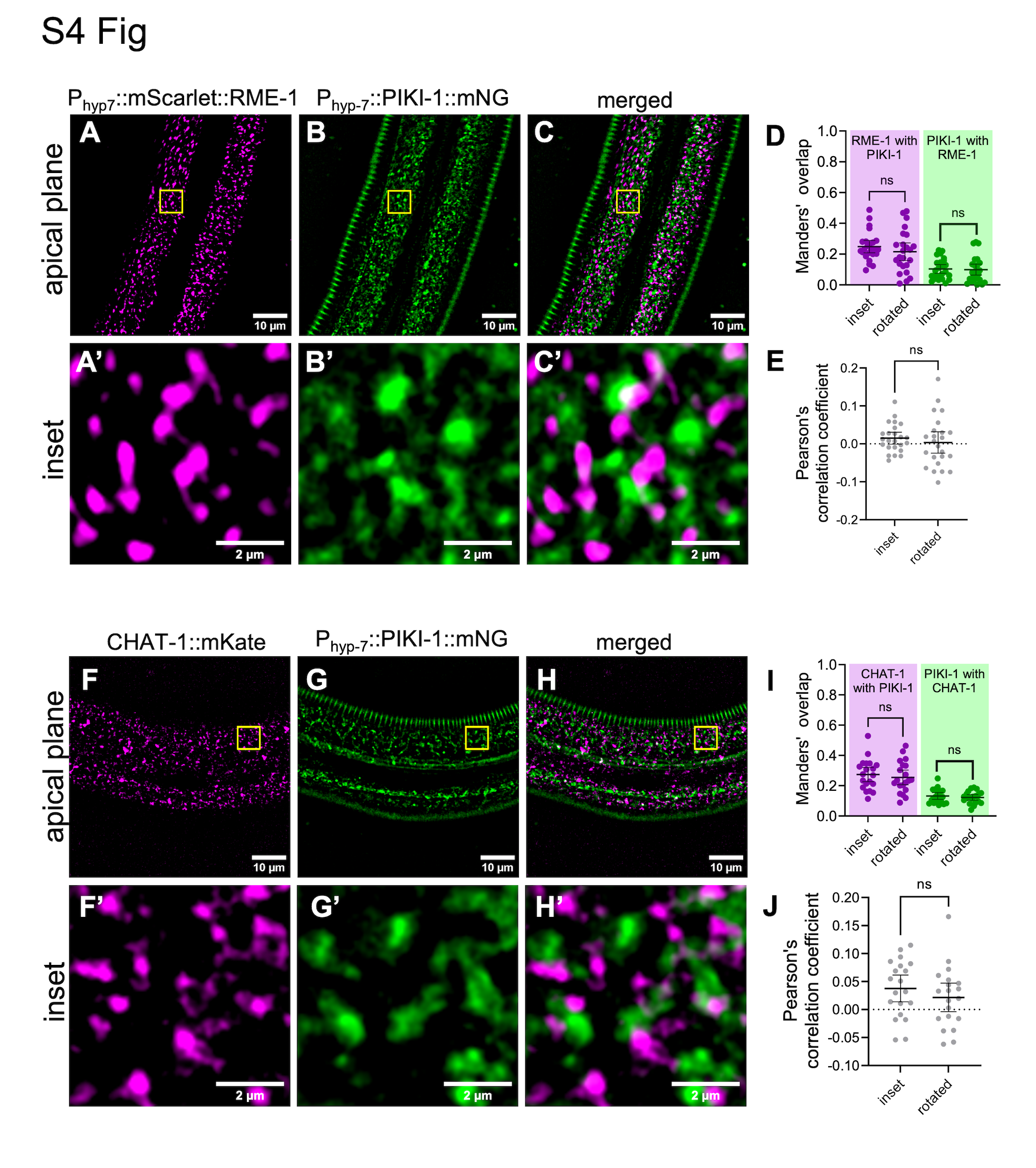

Supplement: S4 Fig — (A–C’ and F–H’) Colocalization of young adult worms expressing heterozygous Phyp7::PIKI-1::mNeonGreen with (A–C’) heterozygous Phyp7::mScarlet::RME-1 (n = 24) or (F–H’) heterozygous CHAT-1::mKate (n = 20). Yellow squares correspond to the enlarged insets; yellow arrows indicate examples of colocalization (white). The seam cell is labeled in A–C and F–H. Colocalization was quantified using Mander’s overlap (D, I) and Pearson’s correlation coefficient (E, J). Dot plots show the mean and 95% CI. Statistical significance between rotated and inset values were determined using unpaired t-tests; ns, not significant. Raw data are available in S1 File. (TIFF) [file pgen.1011740.s004.tiff]

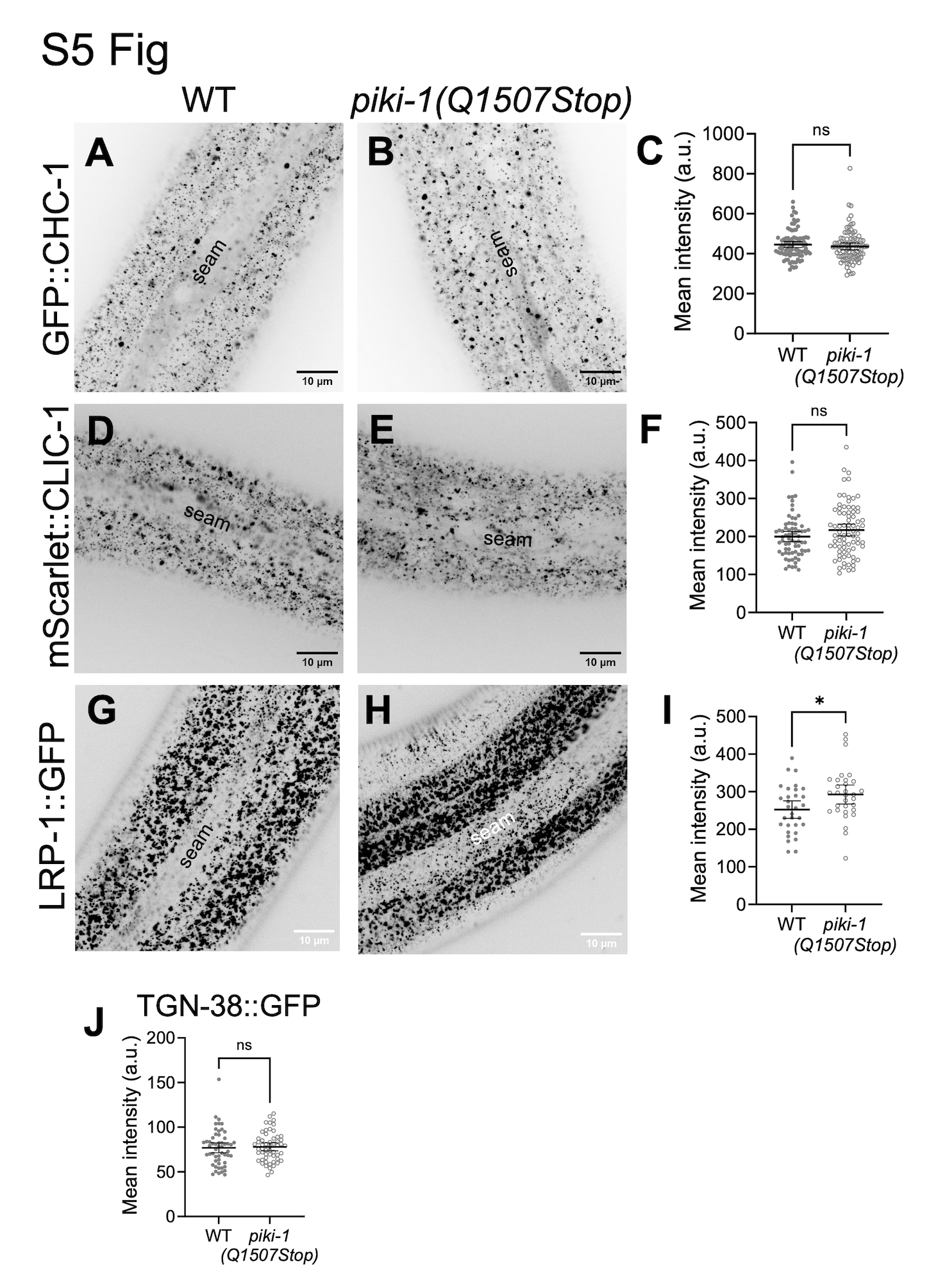

Supplement: S5 Fig — (A, B, D, E, G and H) Representative confocal images of day-1 adults in wild-type and piki-1(Q1507Stop) mutants expressing (A, B) GFP::CHC-1, (D, E) mScarlet::CLIC-1, and (G, H) Phyp7::LRP-1::GFP. The seam cell is labeled in all images. (C, F, and I) Mean intensities corresponding to markers shown in the left-hand panels; (J) Mean intensity for TGN-38::GFP in wild-type and piki-1(Q1507Stop) mutants (also see Fig 3M and 3N). Dot plots show the mean and 95% CI. Statistical significance was determined using unpaired t-tests; *p ≤ 0.05; ns, not significant. Raw data are available in S1 File. (TIFF) [file pgen.1011740.s005.tiff]

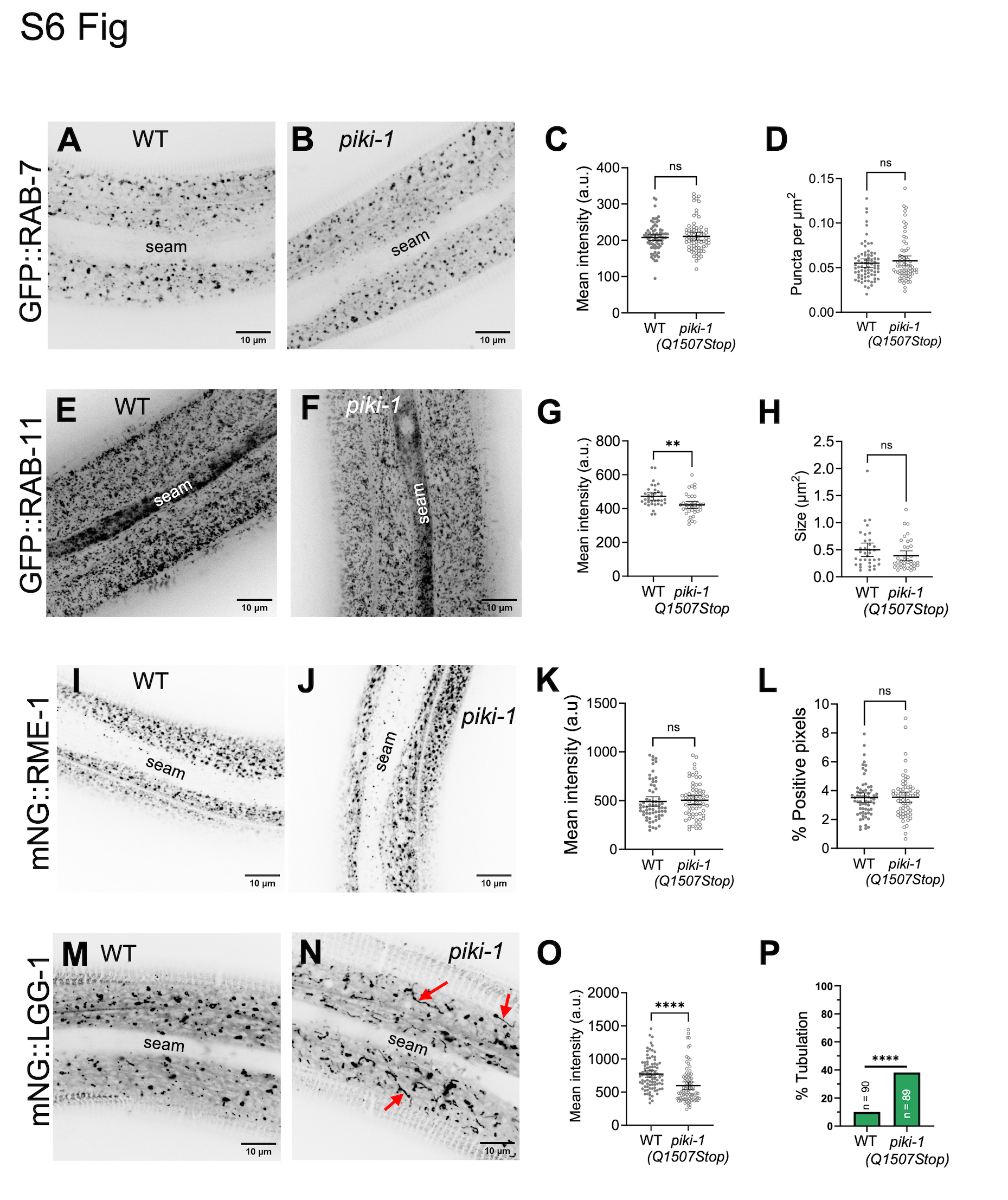

Supplement: S6 Fig — (A, B, E, F, I, J, M and N) Representative confocal images of day-1 adults in wild-type and piki-1(Q1507Stop) mutants expressing (A, B) GFP::RAB-7, (E, F) Phyp7::GFP::RAB-11, (I, J) Phyp7::mNeonGreen::RME-1, (M, N) Phyp7::mNeonGreen::LGG-1. The seam cell is labeled in all images. Red arrows (N) indicate instances of tubulation. (C–P) Indicated parameters corresponding to the left-hand panels are indicated. Dot plots show the mean and 95% CI. Statistical significance (C–O) was determined by an unpaired t-test or (P) Fisher’s exact test. ****p ≤ 0.0001; **p ≤ 0.01; ns, not significant. Raw data are available in S1 File. (TIFF) [file pgen.1011740.s006.tiff]

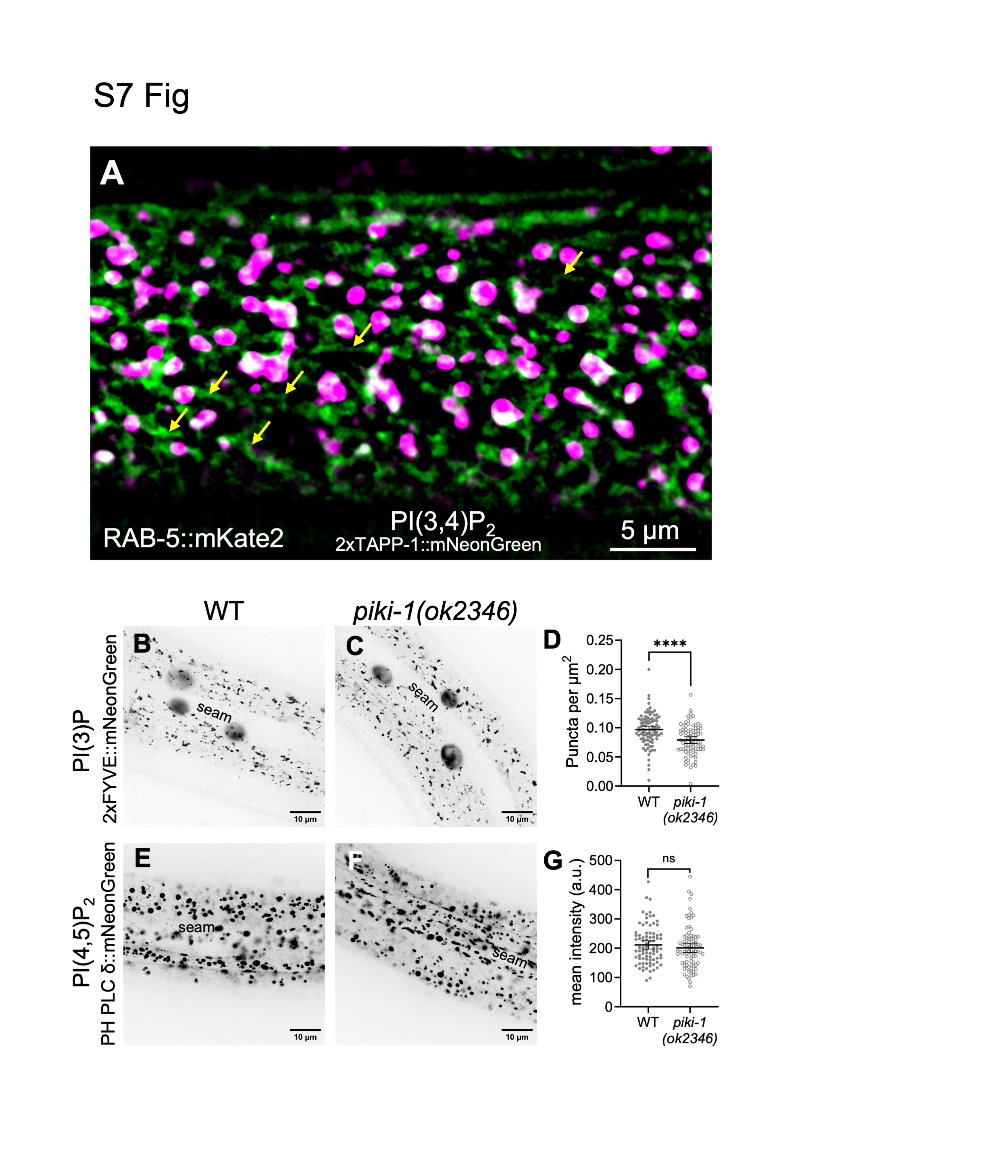

Supplement: S7 Fig — (A) Enlarged image of RAB-5::mKate and 2xTAPP-1::mNeonGreen [PI(3,4)P2] markers showing protrusions and tubule-like structures (green) emanating from early endosomes (magenta). (B, C, E and F) Representative confocal images of wild-type and piki-1(ok2346) day-1 adults expressing (A, B) a PI(3)P lipid sensor [Phyp7::2xFYVE::mNeonGreen] and (D, E) a PI(4,5)P2 lipid sensor [Phyp7::PH PLC δ::mNeonGreen]. (D) Puncta per unit area for worms expressing Phyp7::2xFYVE::mNeonGreen were slightly decreased. (G) Mean intensity for worms expressing Phyp7::PH PLC δ::mNeonGreen was unchanged. Dot plots show the mean and 95% CI. Statistical significance was determined using unpaired t-tests; ****p ≤ 0.0001; ns, not significant. Raw data are available in S1 File. (TIFF) [file pgen.1011740.s007.tiff]

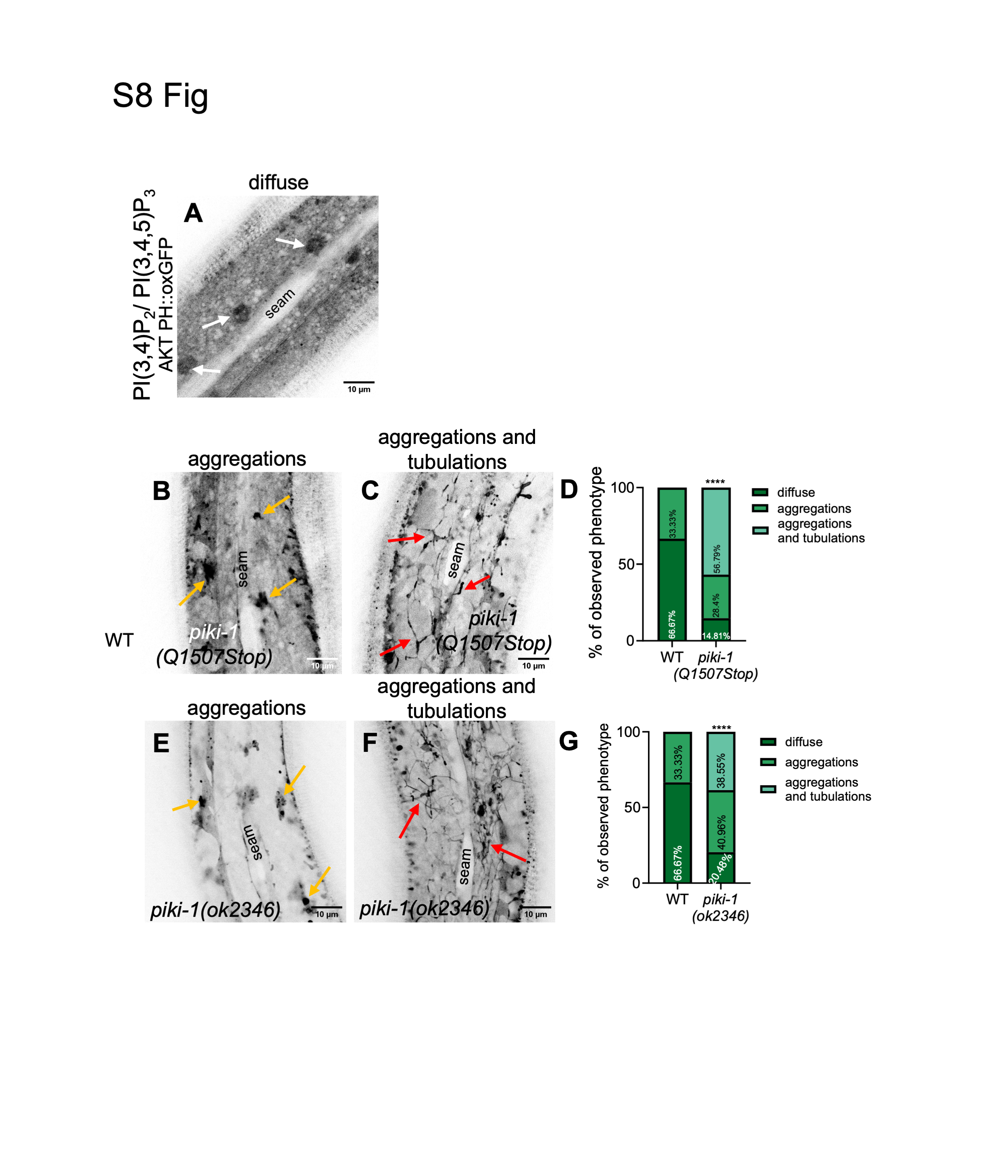

Supplement: S8 Fig — (A–C, E and F) Representative confocal images of day-1 adults expressing the PI(3,4)P2/PI(3,4,5)P3 lipid sensor Phyp7::AKT::oxGFP in (A) wild-type, (B and C) piki-1(Q1507Stop), or (E and F) piki-1(ok2346) backgrounds. White arrows (A) indicate nuclei. Orange arrows (B, E) indicate aggregations. Red arrows (C, F) indicate tubulations. (D, G) Individual worms expressing Phyp7::AKT-PH::oxGFP were scored for the presence of diffuse labeling, aggregations, or aggregations and tubulations within the epidermis. Statistical significance of the differences in phenotype distributions were determined using Chi-squared tests; ****p < 0.0001. Raw data are available in S1 File. (TIFF) [file pgen.1011740.s008.tiff]

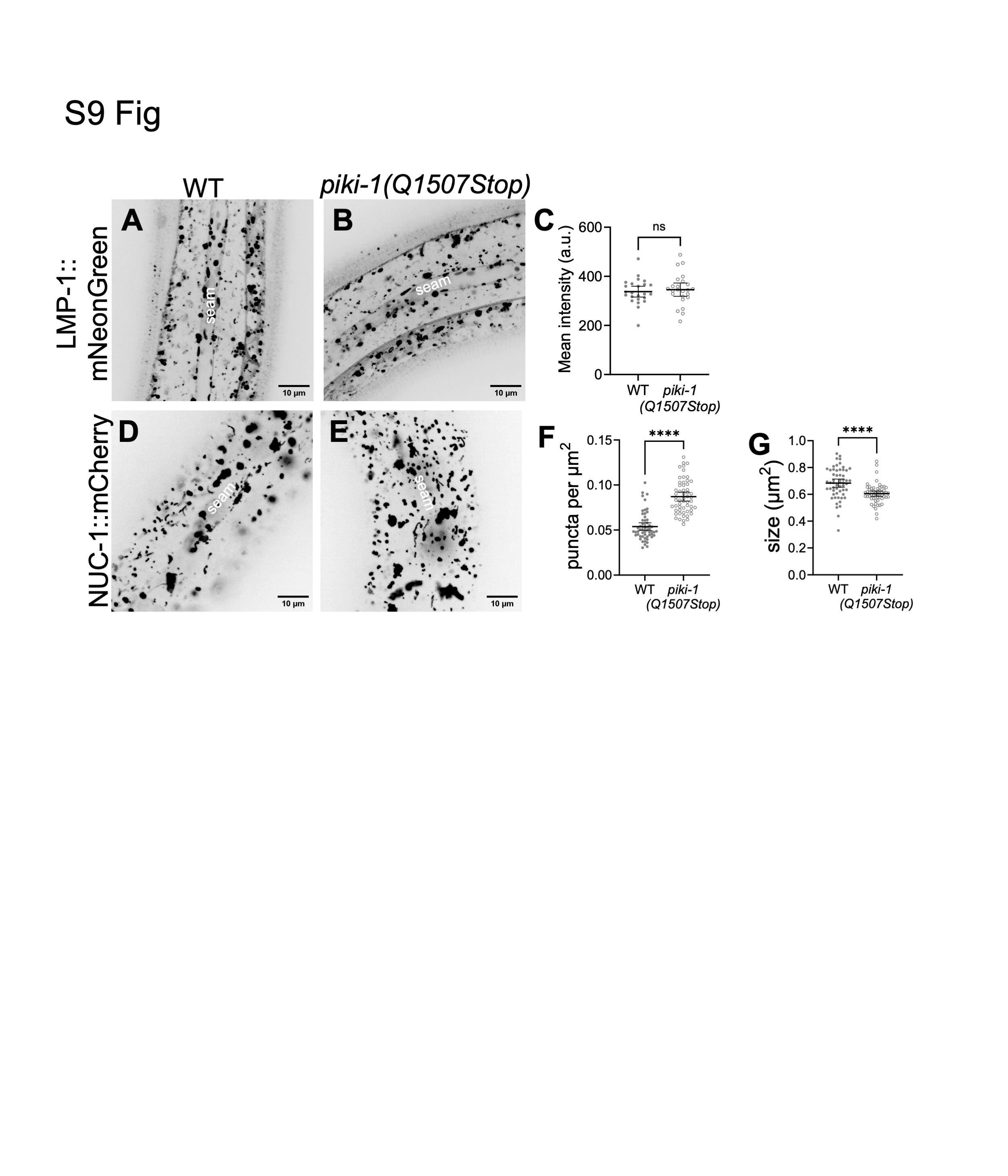

Supplement: S9 Fig — (A, B, D and E) Representative confocal images of day-1 adults expressing (A, B) Phyp7::LMP-1::mNeonGreen or (D, E) NUC-1::mCherry in (A, D) wild-type and (B, E) piki-1(Q1507Stop) backgrounds. (C) Mean intensity was plotted for Phyp7::LMP-1::mNeonGreen. (F) The number of puncta per unit area and (G) size of puncta were plotted for NUC-1::mCherry. Dot plots show the mean and 95% CI. Statistical significance was determined using unpaired t-tests; ****p ≤ 0.0001; ns, not significant. Raw data are available in S1 File. (TIFF) [file pgen.1011740.s009.tiff]

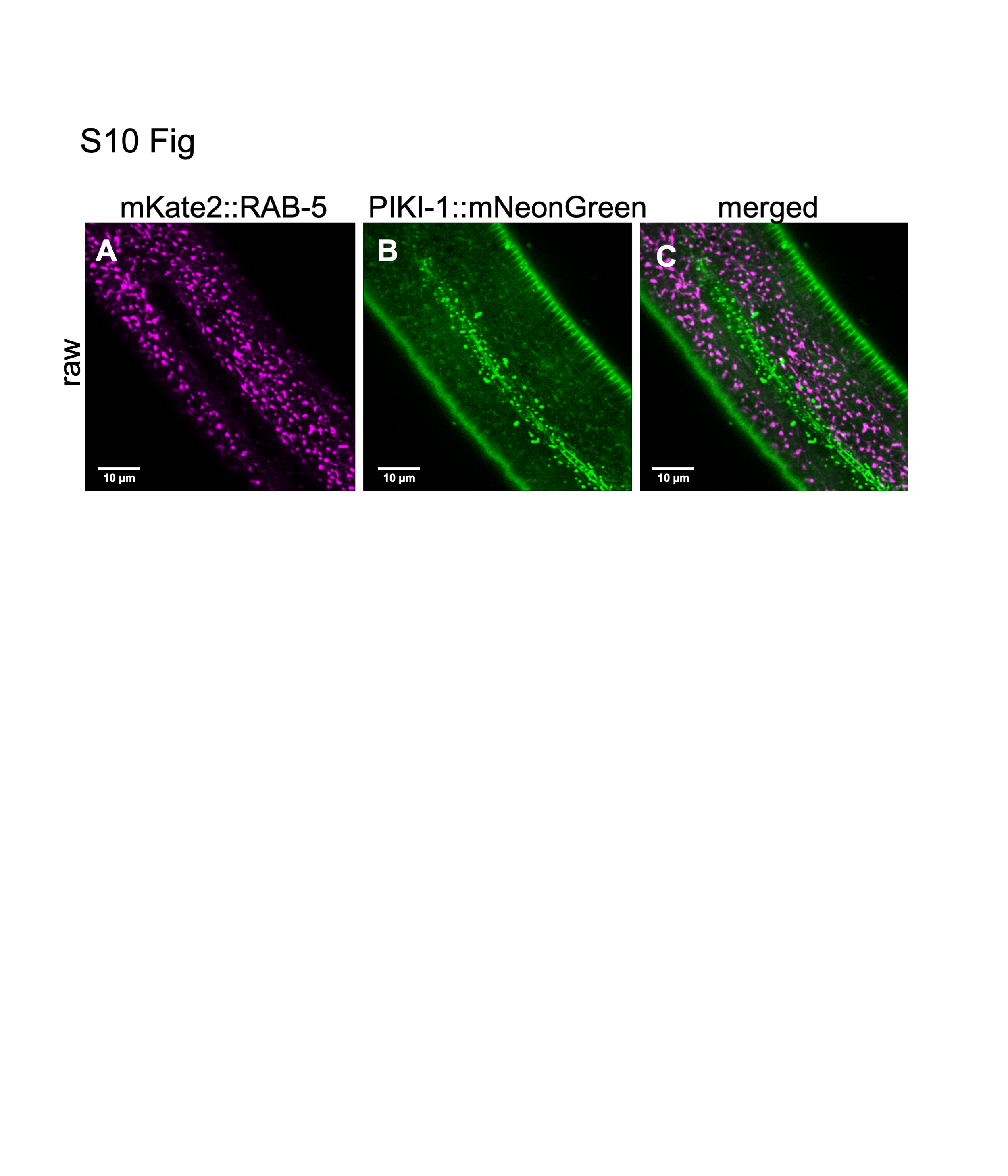

Supplement: S10 Fig — (A–C) Representative raw images of a day-1 adult homozygous for both Phyp7::PIKI-1::mNeonGreen and Pdpy-7::mKate2::RAB-5. Both (A,B) single-channel and (C) merged images are shown. Note abnormal expression of PIKI-1 in the seam cell. (TIFF) [file pgen.1011740.s010.tiff]
