# Supplementary material for: PIKI-1, a class II PI 3-kinase, functions in endocytic trafficking
Source: PLoS Genet. 2026 Feb 13;22(2):e1011740. doi: 10.1371/journal.pgen.1011740 (PMC12919929; doi:10.1371/journal.pgen.1011740)
Supplement: Attachment — Submitted filename: Reimann_Respose_to_reviewers.pdf [file pgen.1011740.s016.pdf]

To the editors of PLoS Genetics:

We sincerely thank the reviewers and editors for their time and input into the manuscript. The reviewer's individual points are addressed below including a number of the requested experiments. In addition, we carried out several experiments not specifically requested that we believe substantially strengthen our manuscript.

This process has taken us more time than usual because the paper's first author started medical school in July and then endured a serious medical condition followed by surgery, from which she is still recovering…

Reviewer #1: Reimann et al. present a rigorous and quantitative study demonstrating important roles of the class II PI3Kinase in endosomal organelles and cuticular molting. Using the power of genetics, they show that loss-of-function mutations in piki-1 suppress the molting defects of mutants that enlarge early endosomes, likely by decreasing the number and size of early endosomes. They also demonstrate that PI3,4P2 levels are altered in piki-1 mutants, suggesting that PIKI-1 is the major kinase acting on PI4P to generate PI3,4P2. This suggests the model that PI3,4P2 lipids influence early endosome size and number, which is further corroborated by the identification of a PI3,4P2-binding protein with similar genetic interactions to PIKI-1. These findings are a significant starting point for identifying how PI3,4P2 influences endosomal trafficking with players conserved across animals.

The authors started to explore the trafficking mechanism further and find that piki-1 mutations increase the number of tubules containing Golgi-PM-cycling TGN-38 and Atg8-like LGG-1. However, these markers are found on a diverse range of organelles, which makes it challenging to determine the step at which PIKI-1 or PI3,4P2 are likely to act. Their data suggests that the tubules are also likely covered in PIP3 and the authors propose that these are early endosomal tubules, but the nature and dynamics of the tubules are unclear due to the absence of two-color or time-lapse imaging. The tubulation findings identify a number of informative avenues to pursue in later research and expose gaping holes in our current knowledge of membrane trafficking, but are difficult to synthesize into a mechanistic model at this point.

Major comments

Line 194 - Do the authors think the premature stop codons in piki-1 are likely to cause nonsense-mediated decay or truncate the C2B domain? NMD could be tested experimentally by checking piki-1 mRNA levels, performing a PIKI-1 western (assuming antibodies exist), or suppressing NMD in their triple mutant strain. Also, it would be helpful to briefly comment on what the C2B domain is thought to do. That could help illuminate which subset of PIKI-1 functions are likely to contribute to nekl rescue.

We examined the question of nonsense-mediated decay by placing suppressed *nekl-2; nekl-3 piki-1(Q1503Stop)* mutants on RNAi plates for *smg-1*, *smg-3*, and *smg-4*. Although we failed to observe de-suppression of molting defects in this strain, our positive control strains [*dpy-5(e61)* and *unc-54(r293)*] also failed to exhibit reversion of their associated phenotypes, which is likely due to insufficient knockdown of the NMD pathway by RNAi. As such, we were unable to draw any conclusions from these experiments. Instead, we have added a line to indicate the possibility of NMD of these alleles (lines 175-177).

We note the suppression of *nekls* by *piki-1* reduction-of-function is strongly supported by the other recessive *piki-1* alleles (including a large deletion). Moreover, we addressed a related

question from Reviewer #2 by generating a catalytically defective *piki-1* mutant using CRISPR and found that it too suppressed *nekl-2; nekl-3* mutants (see below).

To address the question about the C2B domain, which is expected to be absent in the Q1503Stop and Q1507Stop mutant alleles, we have added a brief discussion (lines 173-175).

Line 235 - Given the challenges in making PIKI-1 FP knock-ins with consistent localization patterns, do the authors know if any of their tagged PIKI-1s are functional? Does the mScarlet knock-in suppress nekl-2(-); nekl-3(-) defects like the other loss-of-function alleles? Does PIKI-1::mNG expression in hyp7 reinstate the growth phenotype in nekl-2(-); nekl-3(-); piki-1(-)? Also, could the authors comment on whether the hyp7 promoter is likely to cause PIKI-1 overexpression by comparing published levels of endogenous semo-1 and piki-1 mRNA in the hypodermis?

We ultimately addressed the question of PIKI-1::FP functionality using one of the reviewer's suggested approaches. Namely, we generated a *nekl-2; nekl-3 piki-1::GFP* [CRISRP] strain and failed to observe any suppression of molting defects, indicating that PIKI-1::GFP retains at least partial function (lines 224-226). We also attempted to test the function of the P_{hyp7}::PIKI-1::mNG integrated transgene directly by crossing it into suppressed *nekl-2; nekl-3*; *piki-1* mutants and looking for de-suppression. Unfortunately, the location of the integrated P_{hyp7}::PIKI-1::mNG transgene on LGX precluded this approach as both *nekl-3* and *piki-1* are also on LGX.

With respect to expression levels of *piki-1* versus semo-1(P_{hyp7}), according to WormBase modENCODE data, *semo-1* is expressed at substantially higher levels than *piki-1* during larval stages with ratios ranging from 2.5–5.5-fold and is expressed at >10-fold higher levels in young adults. As such, the transgene is expressed at considerably higher levels than the endogenous gene, consistent with our microscopy observations. We now include a line and references mentioning this (lines 226-229).

Line 366 - To demonstrate a direct role for PIKI-1 in early steps of autophagy, it would be necessary to show significant colocalization of PIKI-1 and LGG-1. This can be rephrased or addressed experimentally. Also, could the LGG-1+ tubules be a sign of CASM rather than autophagosomes? Testing that would require a fair amount of EM tomography to observe whether any tubular structures have single or double membranes. For this study, it may be better to give more open-ended interpretations.

We have re-written this paragraph based on other reviewer comments and have included a more open-ended interpretation regarding the relationship of PIKI-1 to LGG-1-mediated autophagy or phagocytosis in the adult epidermis (lines 351-361).

Line 506 - Given that PIKI-1 is predicted to phosphorylate PI4P, piki-1 mutants may be predicted to have higher levels of PI4P (especially given the observed absence of an increase in PI4,5P2). Have the authors created any PI4P reporters, such as using the P4C domain of SidC? The authors should discuss the possibility that phenotypes could arise from altering PI4P turnover in addition to PI3,4P2 levels. Overexpressing a PI3,4P2 phosphatase or the PI5Kinase required to make PIP3 could also help distinguish between the roles of the different lipids.

This is a good point, and we now discuss this possibility (lines 460-462 and 512-516).

A major challenge in studying trafficking is its cyclical nature, where it is difficult to tell if an increased number of tubules in a static image is due to increased formation of tubules or decreased vesicle release from tubules (longer-lived tubules). Can the author's design any experiments to distinguish between increased tubulation and stalled tubules, i.e. time-lapse imaging or disrupting tubulation factors? Furthermore, comparing tubulation markers would be helpful to distinguish whether the tubulation of multiple organelles is altered or whether everything is likely to be happening on a Rab5+ early endosome.

These are good questions, but somewhat beyond the scope of the current manuscript. We did image tubules in WY2197 [P_{hyp7}::*tgn-38::GFP; piki-1(fd399)*] worms, which we found were largely stable over a period of 5 minutes.

Minor comments
Line 65 - PIP2 should be bisphosphate, not bi

Corrected.

Line 86 - "via PI(4,5)P2" may be getting into too much detail when introducing the formation of PI3,4P2 by class II PI3Ks. If the authors want to introduce PI4,5P2 conversion to PI4P, that would need a separate sentence, but it could also be omitted here for clarity on PI3Ks.

Corrected as suggested.

Fig. 1B - This diagram is very helpful, but would it be possible to move the PI & PI4P entries up to make it clear that Class II PI3Ks are not phosphorylating PI to PI4P? Especially after B&W printing, the green color code will be lost to the reader. With both class II and class III PI3Ks on the same arrow, it gets crowded if the vertical spacing is left symmetric as shown.

This is a good suggestion, and we have shifted PI and PI4P up in the figure for clarity.

Line 165 - Missing a period before "The"

Corrected.

Line 196 - Can the authors speculate on why the S211N mutation would disrupt PIKI-1 function? Is it conserved among this class of PI3Kinases? Otherwise, it would be helpful to highlight in the text that it is in an uncharacterized domain if there is no published supporting data.

We have now added a brief statement regarding this mutation on lines 177-178.

Line 277 - Localization is not equivalent to function, so the conclusion about localization data should be toned down to reflect similar localization between worms and mammals. These data raise the possibility that the function is also conserved, but "suggests the role is conserved" sounds too strong. Also, it would be helpful to add a sentence emphasizing that your data

suggests PIKI-1 also localizes to unidentified discrete structures that are CLIC-1-negative and RAB-5-negative. These could be the LGG-1+ structures.

We now include a comment addressing the likelihood of additional PIKI-1 localization sites (lines 257-258). In addition, prompted by Reviewer #2, we also carried out colocalization studies of PIKI-1 with five additional markers as described below.

Line 290 - “only slightly altered” is vague. Could the authors describe more specifically what they observed?

We have changed this to “showed only slightly increased accumulation at the apical surface” (lines 282-284).

Fig. 3A - Autophagosomes have double membranes, but the diagram shows a single membrane associated with LGG-1. Atg8 family proteins can also be conjugated to single membranes, aka CASM, LANDO, or LAP. Which do the authors think they are observing?

This is good question. At this point we can’t know the precise nature of the LGG-1 marked compartment(s), a point raised by another reviewer. We now acknowledge this ambiguity in the text (lines 357-358).

Fig. 3 Legend - lines 299 & 304 - RAB-7 is missing the FP used, presumably GFP::RAB-7.

Corrected.

Line 349 - It would be helpful to better distinguish between the Golgi markers when introducing AMAN-2, for example referencing what is known about the sub-Golgi localization of alpha-mannosidase II in mammalian cells and emphasizing that AMAN-2 is a Golgi resident protein. It would also be helpful to comment on the lack of AMAN-2+ tubules in piki-1 mutants to better contrast with the TGN-38 findings.

These are good points, which we attempt to better clarify (lines 338-349 and 538-547).

Fig. 4 - Do the authors think that piki-1 is specifically suppressing nekl-2 defects and not nekl-3 defects? The authors interpret these results in terms of organelle specificity, but is it possible to test suppression of individual Nekl phenotypes beyond organelle size?

We sought to test this by generating strains with our moderate loss-of-functional alleles for *nekl-3(sv3)* and *nekl-2(fd91)* with *piki-1(Q1503Stop)*. Although we were unsuccessful in generating the *nekl-3(sv3) piki-1* strain (in part because of a chromosomal conflict), we did obtain a *nekl-2(fd91); piki-1* strain and found that it did not suppress molting defects. Our data suggest that similar to several other *nekl* suppressors, *piki-1* is capable suppressing synthetically lethal double mutants but not stronger loss-of-function alleles. This result is now mentioned (lines 190-193).

Line 469 - missing 2 on PI3,4P2.

Corrected.

Line 481 - which movie?

Corrected, S2.

Fig. 5 - Are PI4P or PI3,4P2 levels altered in nekl-2 or nekl-3 mutants?

This is a very good question, but we have not tested this in the current study.

Line 536-537 - provide full names for SESTD1 & HIP1 for better context

We have defined the names of these proteins.

Line 538 - It's important to repeat the caveat on RNAi when interpreting the negative result with F55D12.2 and contrasting it with hipr-1 RNAi. Otherwise, it gives the impression that F55D12.2 has been ruled out.

We have now included a brief statement about this important caveat, including for other RNAi-based experiments (line 494-496).

Line 565 - Given the AKT-PH tubules in piki-1 mutants, isn't it more likely that either/both PI3,4P2 and PIP3 are enriched on tubules?

We see the reviewer's point but feel uncomfortable making conclusions regarding the lipid composition of the tubules. Namely, we didn't see tubules with the (presumably) more specific PI(3,4,5)P3, PI(3,4)P2 or PI(3)P sensors in *piki-1* mutants, which would have been expected if these lipids were a component of the tubules. We also added a line underscoring our uncertainty about what the AKT sensor may be binding (lines 464-465).

Reviewer #2: General Assessment

This study uses a forward genetic screen in C. elegans to reveal that loss-of-function mutations in piki-1, which encodes the class II PI3K PIKI-1, suppress molting defects associated with NEKL kinases. The authors identify PIKI-1 as a primary regulator of PI(3,4)P_2 in the epidermis and demonstrate that it controls endocytic trafficking by influencing early endosome morphology and protein recruitment. The work is founded on a strong experimental design, and the conclusions are generally well-supported by the data.
However, the manuscript's impact could be significantly enhanced with a more rigorous investigation of the underlying mechanisms, the inclusion of more quantitative analytical methods, and greater clarity in the presentation. The following revisions are recommended to

strengthen the manuscript's claims.

Major Comments

1. The manuscript suggests that Class I PI3K is not involved in generating the relevant $PI(3,4)P_2$ pool. While the data are consistent with PIKI-1 being the primary source, the evidence presented may not be sufficient to definitively exclude any role for Class I PI3K. I recommend moderating this conclusion

We agree and have now added statements to this effect (lines 530-536).

2. Defining the Subcellular Localization of PIKI-1: The study localizes PIKI-1 to RAB-5/CLIC-1-positive early endosomes. However, relying on morphology and colocalization with only early endosome markers is insufficient to rule out its presence in other compartments, especially given the resolution of confocal microscopy. To more precisely define PIKI-1's site of action, I recommend expanding the colocalization analysis to markers for other key organelles in the endo-lysosomal pathway, such as: Late endosomes, Recycling endosomes, Lysosomes. This will provide a more comprehensive map of PIKI-1's localization and strengthen the claims about its specific role in early endocytic events.

This is a good suggestion, and we have now carried out additional co-localization studies with five additional endomembrane markers including GFP::RAB-7 (late endosomes), NUC-1::mCherry (lysosomes), mScarlet::RME-1 (RME-1-positive recycling endosomes), and CHAT-1::mKate (early endosomes and CHAT-1::mKate-positive recycling endosomes) and SNX-1 (early endosomes). Notably, the only marker where we observed clear co-localization was with SNX-1.(S3 Fig, S4 Fig, S7 Fig, and associated text).

In further studies, in *piki-1* mutants mScarlet::RME-1 localization was largely unaltered, but we observed a clear increase in the size of the SNX-1 compartment (Fig 3J–M, S6 Fig, and associated text). Our findings suggest that loss of PIKI-1 function and the commitment reduction in PI(3,4)P2 alters the composition of proteins that bind to early endosomes.

We also recognize that our observed colocalization of PIKI-1 with early endosomes (RAB-5 and SNX-1) and clathrin-coated pits (CLIC-1) likely does not fully account for the distribution of our PIKI-1 marker within cells and have added statements to this effect (lines 257-258 and 270-271).

3. A central conclusion is that piki-1 mutations impair the enzyme's phosphotransferase activity, leading to reduced $PI(3,4)P_2$. The current evidence is based on genetic suppression, which is indirect. The observed phenotypes in the truncated and missense mutants could result from protein instability rather than a specific defect in catalysis.

(Also see Reviewer #1 comment #1) To address this question, we generated a catalytically defective *piki-1* mutant strain using CRISPR in *nekl-2; nekl-3* (and N2) genetic backgrounds. We find that *piki-1(D1189A)* indeed suppresses *nekl-2; nekl-3* molting defects, indicating that a reduction or loss of phospho-transferase activity is sufficient for suppression and that the catalytic activity of PIKI-1 is relevant to its genetic interaction with the *nekls* (lines 185-188 and Fig 1F). We also mention protein stability via NMD pathways (lines 175-177).

4. The study establishes a clear genetic relationship between PIKI-1 and NEKL, but the molecular nature of this interaction remains unclear. Is there any physical interaction between PIKI-1 and NEKL? Assess whether loss of piki-1 affects NEKL kinase activity or localization, which would help position the two proteins relative to one another in the signaling pathway.

We have now added some additional discussion regarding the potential for more direct interactions of PIKI-1 with NEKL–MLT proteins (lines 570-573). Namely, we cite data from our recent proximity labeling study (PMID: 41381733) showing that PIKI-1 was significantly enriched in two independent NEKL-2::TurboID experiments. This result suggests that NEKL-2 may directly or indirectly interact with PIKI-1, although it is also possible that their co-localization to early endosomes may account for this result. As an aside, we used AF2 and AF3 to look for a possible direct interaction between NEKL-2 and PIKI-1 (full-length PIKI-1 and individual domains) but did not obtain strong evidence. Further studies will be necessary to obtain a detailed mechanistic understanding.

5. The hypothesis that PI(3,4)P_2 reduction suppresses nekl defects via the effector protein HIPR-1 is compelling but requires more direct evidence. The logical chain connecting PIKI-1, PI(3,4)P_2, and HIPR-1 is currently incomplete.

We agree and have weakened our previous statements (lines 30-34, 494-500).

Minors

1. Logical Flow & Focus: The transition to Class II PI3Ks (line 83) feels abrupt. Please add a sentence to frame why this class is understudied. I also suggest condensing the descriptions of Class I/III PI3Ks to expand on the known roles and open questions regarding Class II PI3Ks, better highlighting the novelty of your work. Rationale: Please explicitly state the rationale for focusing on PIKI-1.

We agree with this comment and have extensively re-worked much of the introduction (lines 48-123) to better frame our studies on PPIns and Class II PI3Ks. The rationale for studying PIKI-1 comes somewhat later in the introduction (starting line 138) after we introduce the *nekls* and the findings of our suppressor screen.

2, Citations issues

(Lines 82-87): For the role of PI(3,4)P_2 in endocytic recycling, please cite more direct evidence, such as Roman-Fernandez et al. (ref 51).

Thank you for pointing this out. The citation issues have been addressed as requested.

(Lines 67-71): If available, add key foundational references regarding PI(4,5)P_2's role in clathrin-mediated endocytosis.

The citation issues have been addressed as requested.

3. (Line 326): The data show changes in endosome morphology, not function. Rephrasing "role in the function of early endosomes" to "role in the biogenesis or homeostasis of early endosomes" would be more accurate.

Corrected (lines 299-300).

(Line 335): The term "only a minor or indirect role" is too definitive. Removing "only" creates a more appropriately cautious statement.

Corrected.

(Line 291): Remove "largely" for a more direct statement.

Corrected.

(Line 363-364): The phrase "although the effect on LGG-1 was less robust" is an unsupported comparison and should be removed or statistically substantiated.

We agree and re-written much of the relevant paragraph S6M–P Fig (351-361).

4. Fig S7: The nNeonGreen signal driven by the Phyp7 promoter appears in seam cells. Please clarify if this is expected and whether it might affect colocalization analysis.

This result was indeed not expected but as now stated more clearly in the Materials and methods (lines 680-687) suggest that this could be the result of a genetic interaction caused the tags or from an overexpression artifact, either of which could alter the normal expression of the P_{hyp7}::mNG::PIKI-1 marker. For this reason, we carried out co-localization of PIKI-1::mNeonGreen and mKate2::RAB-studies in animals that were heterozygous for both markers. Importantly, the expression of P_{hyp7}::mKate::RAB-5 and P_{hyp7}::mNG::PIKI-1 in heterozygous worms looked similar or identical to the expression observed in the parental homozygous marked strains. We note that we carried out several of our co-localization studies in trans-heterozygous strains for convenience (lines 680-681) but did not test for anomalies in the homozygous lines. We have included this result to alert readers to potential functional interactions for markers of this nature (S11 Fig), although it could be removed.

We also tried several additional approaches including the generation a CRISPR-tagged mKate::RAB-5 but found that this strain was lethal as a homozygote. Nevertheless, the expression of (CRISPR) mKate::RAB-5 in heterozygous worms appeared similar or identical to P_{hyp7}::mKate::RAB-5 (data not included in this paper). We also note the observed co-localization of SNX-1 with RAB-5 and PIKI-1, consistent with localization of all three proteins to early endosome compartments (see above and S7 Fig). Also, as mentioned, the expression of CRISPR generated PIKI-1::GFP was too low to be useful in our studies. In summary, we did due diligence to test the co-localization of PIKI-1 with RAB-5 and early endosomes by taking the steps outlined above.

5. Please standardize the font sizes of labels within figures for consistency and a more professional appearance (e.g., Fig 3 H, I, L, M; Fig S3 A-F).

We appreciate the reviewer catching that detail. We have gone through all our figures to ensure greater consistency of font sizes etc.

6. Please add the full strain names, in addition to allele names to ensure reproducibility.
The complete strain names and genotypes are now included in S2 File.

Reviewer #3: Summary:

Two conserved NIMA127 related Ser/Thr protein kinases, NEKL-2 (NEK8/9 in mammals) and NEKL-3 (NEK6/7), and a number of their binding partners, are essential for proper molting via the regulation of endocytic trafficking. In nekl-2; nekl-3 double mutants, molting is arrested. These mutants suffer 98% larval lethality. Through a well-designed forward genetic screen, the authors isolated a number of extragenic suppressor mutants of the synthetic lethal phenotype. Among the suppressor mutants there are three loss-of-function alleles of piki-1, which encodes the only Class II PI-3 kinase in C. elegans. This manuscript reports the function of PIKI-1 in endocytic recycling in hypodermal cells. The authors found that the primary activity of PIKI-1 is to produce PI(3,4)P2 on the surfaces of early endosomes. Consistent with this activity, PIKI-1 is localized to clathrin-associated compartments and to early endosomes. In piki-1(lof) mutants, among a number of endocytic-related vesicles, early endosomes are specifically abnormal. Particularly, there are fewer early endosomes, and these endosomes are smaller. There are also other phenotypes related to endosomes, such as the tubulation phenotype. Regarding the functional relationship between PIKI-1 and NEKL-2/-3, the suppression of the lethal phenotype displayed by the nekl-2; nekl-3 double mutants by the lof mutations of piki-1 indicates that PIKI-1 acts downstream of NEKL-2 and NEKL-3 and antagonizes their functions. In support of this conclusion, piki-1 mutations appear to suppress the early endosome number and morphological defects displayed by nekl-2 mutants (yet this reviewer has some questions regarding the impact of the opposite early endosome defects displayed by piki-1 and nekl-2 single mutants (see below – Major Concerns)). Furthermore, inactivation of HIPR-1, a potential PI(3,4)P2-binding protein, partially suppresses the larval lethality of the nekl-2; nekl-3 double mutants. This result support the model that “reduced levels of PI(3,4)P2 on early endosomes, along with an accompanying reduction of PI(3,4)P2-binding proteins such as HIPR-1, lead to the suppression of nekl-2; nekl-3 molting defects and to the partial restoration of trafficking functions”.

Evaluation:

This manuscript reveals, for the first time, the potential function of PI(3,4)P2 in an essetial developmental process. It also reveals that the primary function of PIKI-1 is to produce PI(3,4)P2 on early endosomes. Unlike Classes I and III PI-3 kinases, Class II PI-3 kinases are not as well characterized. Class II PI-3 kinases are implicated in vesicle trafficking, cell signaling, and cell migration events. C. elegans PIKI-1 is known to facilitate the degradation of engulfed apoptotic cells confined inside phagosomes by producing PI(3)P in addition to Class III PI-3 kinase VPS-34. The report that PIKI-1’s function in early endosomes by the authors further discovers additional functions of PIKI-1 in cellular functions. The suppression of nekl-2; -3 double mutants further discloses the function of PIKI-1 in development. These novel discoveries should be shared with researchers studying phospholipid, intracellular trafficking, cell signaling, and animal development. This manuscript will be interested by a broad audience. That said, there are a few weak or unclear aspects that needs clarification and/or improvement. These aspects are described below.

Major concerns:

1. The molecular mechanisms behind the functional relationship between the NIMA127 related Ser/Thr protein kinases NEKL-2 and -3 and PI-3 kinase PIKI-1:
The suppression effects of PIKI-1 strongly suggest that NEKL-2/-3 act to inhibit the activity of PIKI-1. what is the molecular mechanism behind this inhibitory effect? This is a challenging question and there are many aspects to explore. For one of those aspects, which is to determine whether this inhibitory functions of the NEKL proteins targets PI(3,4)P2 on early endosomes, the authors should test:
(1) in nekl-2/-3 mutants, whether the level of PI(3,4)P2 increases on early endosomes.
If NEKL-2/-3 act as inhibitors of the PI-3 kinase activity of PIKI-1, one would expect that inactivating these upstream inhibitors will result in more PIKI-1 products (PI(3,4)P2) on early endosomes.

These are all very good ideas, some of which were raised above. In short, we recognize the utility of such experiments, but they are beyond our current ability to carry out in a timely manner. In the discussion section, we now reference a result from our published proximity labeling study showing that proximity labeling using NEKL-2::TurboID identified PIKI-1 in two independent experiments, suggesting a potential interaction, however, we have no current evidence for a direct interaction or direct regulatory connection (lines 570-573).

(2) whether HIPR-1 binds PI(3,4)P2.
HIPR-1 is a proposed PI(3,4)P2-binding protein. However, whether this is the case has not been tested. The HIPR-1/PI(3,4)P2 binding ability needs to be tested in vitro and/or in vivo, in order to determine whether the proposed model is correct.

We understand the reviewer's point and, as stated above, we have altered statements regarding HIPR-1 to better reflect the level of our evidence (lines 30-34, 497-500, 564-566).

2. How are the defects in the morphology and amount of early endosomes related to the developmental defects.
-- In piki-1(lof) mutants, the authors report that early endosomes are smaller and fewer in number than wild-type. In nekl-2(-) mutants, the authors have observed that early endosomes are bigger and more in number than wild-type, and the early endosomal report displays a brighter intensity. Some clarification is needed as to which (or both) of the opposite phenotypes displayed by the piki-1(-) and nekl-2(-) single mutants will impair the efficiency of endosomal trafficking. Does the presence of larger and more early endosomes indicate an arrest of the proper processing of early endosomes? Does the reduction of early endosome size and number indicate a reduction of early endosome biogenesis? The impacts of these anomalies have not been discussed. Also, it seems that, although piki-1(-) suppresses both the endosomal morphology and amount defects and the larval lethality, this report still has not firmly established that early endosomal defects is the cause for the molting defect of the nekl-2; -3 mutants. The authors need to discuss these issues and other possible models.

These are all good questions, some of which we now address in the Discussion in conjunction with our new findings (e.g., lines 506-514, 527-536, 538-547, 554-556, 562-566, 568-579). It's important to point out, however, that we don't have a precise answer to most of these questions, although some speculation seems reasonable within the Discussion to tie our findings together.

3. The molting defect occurs during larval development, yet the characterization of PIKI-1 subcellular localization and the effect of the piki-1 mutations in the signal intensity of a number of fluorescent reporters were performed in one-day old adults. What are the results of the cell biological assays in larval stages? Are they the same as what have been observed in adults?

We use the auxin-inducible-degron system as a means for assessing the effects of NEKL loss on epidermal cell biology independent of concurrent molting defects (PMID: 32069276). From our previous work, we know that a failure to molt can lead to secondary effects on the cell biology and physiology of worms. For this reason, we routinely inactivate NEKLs in post-molting (young adults) animals to avoid this complication and to allow for the separation of molting versus trafficking defects. Additionally, the quality of imaging is considerably improved in young adult worms than in smaller larvae.

Nevertheless, our earlier (pre-AID system) studies did examine a subset of membrane compartment markers in *nekl-2(RNAi)* (dsRNA-injected) worms. In some cases, these defects were similar (though more variable) to the effects we observe using the AID system; For example, *nekl-2(RNAi)* led to an increase in the size of RAB-5::GFP puncta in some animals (PMID: 25523392).

Specific concern:

Discussion, lines 590-594:
"Previous studies in C. elegans embryos and in the adult gonad indicate that PIKI-1 has a primary role in autophagy in these contexts [72,74–76]. For example, PI(3)P production, which controls autophagy in the C. elegans embryo, is dependent on the coordination among PIKI-1, VPS-34, and MTM-1 (a 3-phosphatase). In addition, loss of PIKI-1 in the embryo and in the gonad strongly impairs autophagosome clearance [76]".

--None of references 72, 74, 75, 76 indicates that PIKI-1 plays a primary role in autophagy. Specifically, reference 76 reports that loss of PIKI-1 function strongly impairs the clearance of phagosomes that contain apoptotic cells, not autophagosomes. The authors seem to mix up autophagosomes, which are ER-originated intracellular organelles, with phagosomes, which are a vacuole produced by engulfment.

We greatly appreciate the reviewer pointing this out these errors and have corrected our mistakes including the suggested corrections to Fig 3A (lines 351-361, 549-556). We note that there is considerable overlap in the proteins required for autophagy and the processing of phagocytosed cell corpses based on prior publications, which the reviewer is likely aware of.

Editor:
Additional minor comment: Could the authors comment on the 'ectopic' signal appearing in the seam of fig. S7? Is it related in any way to the signal in the seam in fig. 5H?

This is a good catch that was also made by Reviewer #2, which we have addressed above (R2; point 4, minor concerns).
